# A novel hybrid aspirin-NO-releasing compound inhibits TNFalpha release from LPS-activated human monocytes and macrophages

**DOI:** 10.1186/1476-9255-5-12

**Published:** 2008-07-31

**Authors:** Catriona M Turnbull, Paolo Marcarino, Tara A Sheldrake, Loretta Lazzarato, Clara Cena, Roberta Fruttero, Alberto Gasco, Sarah Fox, Ian L Megson, Adriano G Rossi

**Affiliations:** 1 Centre for Cardiovascular Science Queen's Medical Research Institute, University of Edinburgh, Edinburgh, EH16 4TJ, UK; 2 Dipartimento di Scienza e Tecnologia del Farmaco, Università degli Studi di Torino, Turin, Italy; 3 Free Radical Research Facility, UHI Millennium Institute, Inverness, IV2 3BL, UK; 4 MRC Centre for Inflammation Research, Queen's Medical Research Institute, University of Edinburgh, Edinburgh, EH16 4TJ, UK

## Abstract

**Background:**

The cytoprotective nature of nitric oxide (NO) led to development of NO-aspirins in the hope of overcoming the gastric side-effects of aspirin. However, the NO moiety gives these hybrids potential for actions further to their aspirin-mediated anti-platelet and anti-inflammatory effects. Having previously shown that novel NO-aspirin hybrids containing a furoxan NO-releasing group have potent anti-platelet effects, here we investigate their anti-inflammatory properties. Here we examine their effects upon TNFα release from lipopolysaccharide (LPS)-stimulated human monocytes and monocyte-derived macrophages and investigate a potential mechanism of action through effects on LPS-stimulated nuclear factor-kappa B (NF-κB) activation.

**Methods:**

Peripheral venous blood was drawn from the antecubital fossa of human volunteers. Mononuclear cells were isolated and cultured. The resultant differentiated macrophages were treated with pharmacologically relevant concentrations of either a furoxan-aspirin (B8, B7; 10 μM), their respective furazan NO-free counterparts (B16, B15; 10 μM), aspirin (10 μM), existing nitroaspirin (NCX4016; 10 μM), an NO donor (DEA/NO; 10 μM) or dexamethasone (1 μM), in the presence and absence of LPS (10 ng/ml; 4 h). Parallel experiments were conducted on undifferentiated fresh monocytes. Supernatants were assessed by specific ELISA for TNFα release and by lactate dehydrogenase (LDH) assay for cell necrosis. To assess NF-κB activation, the effects of the compounds on the loss of cytoplasmic inhibitor of NF-κB, IκBα (assessed by western blotting) and nuclear localisation (assessed by immunofluorescence) of the p65 subunit of NF-κB were determined.

**Results:**

B8 significantly reduced TNFα release from LPS-treated macrophages to 36 ± 10% of the LPS control. B8 and B16 significantly inhibited monocyte TNFα release to 28 ± 5, and 49 ± 9% of control, respectively. The B8 effect was equivalent in magnitude to that of dexamethasone, but was not shared by 10 μM DEA/NO, B7, the furazans, aspirin or NCX4016. LDH assessment revealed none of the treatments caused significant cell lysis. LPS stimulated loss of cytoplasmic IκBα and nuclear translocation of the p65 NF-κB subunit was inhibited by the active NO-furoxans.

**Conclusion:**

Here we show that furoxan-aspirin, B8, significantly reduces TNFα release from both monocytes and macrophages and suggest that inhibition of NF-κB activation is a likely mechanism for the effect. This anti-inflammatory action highlights a further therapeutic potential of drugs of this class.

## Background

Aspirin (acetylsalicylic acid) was first synthesized in 1899 and was the first example of the family of nonsteroidal anti-inflammatory drugs (NSAIDs). Its therapeutic uses include the treatment of headache, rheumatic pain and inflammation, in addition to being utilised as an effective prophylactic against thrombotic events in the cardiovascular system. The anti-inflammatory effects of aspirin are achieved primarily through inhibition of cyclooxygenase-mediated synthesis of pro-inflammatory prostanoids [1]; it causes irreversible inhibition by selectively and rapidly acetylating a serine residue (Ser 530) near the C-terminus of the cyclooxygenase (COX) family of enzymes, forming an impediment to the binding of arachidonic acid [2-4]. The acetylation evokes a requirement for new COX to be synthesised for subsequent production of prostaglandins. Unfortunately, gastrointestinal disorders, including ulceration, are a common side-effect of aspirin, limiting its long-term use [5-8]. The effect is primarily believed to be due to inhibition of the production of prostaglandins that normally protect the gastric mucosa [8-11].

**Figure 1:**
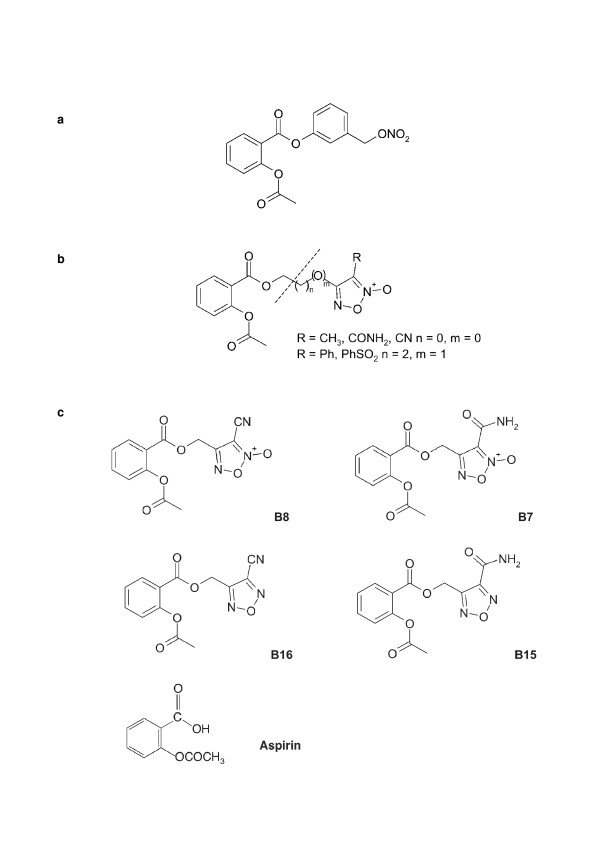
**Structural formulae: a**. The structural formula of NO-aspirin, NCX4016.  **b**. The general structural formula of a furoxan-aspirin hybrid drug. **c**. The structural formulae of furoxans (B8 and B7), their NO-free equivalents, the furazans (B16 and B15) and, for comparison, aspirin.

Aspirin esters containing a nitric oxide (NO)-donor moiety overcome the gastric side-effects [12,13] likely via the cytoprotective effects of drug-derived NO. NO increases blood flow in the gastric mucosa, promoting repair and removal of toxins [14]. NO also increases secretion of protective gastric mucus [15] and is thought to promote healing of gastric ulcers by promoting angiogenesis [16]. Alternatively, or in addition, the protective effects of NO-aspirin could be due to masking of the carboxylic acid moiety by the ester function [12,17]. Two main subtypes of NO-aspirins have so far been developed: the nitrooxy ester (organic nitrate) derivatives and the furoxan derivatives. The first NO-aspirin hybrid drugs to be synthesised, the NicOx^® ^compounds, NCX4016 (3-(nitroxymethyl)phenyl 2-(acetoxy)benzoate; Fig. 1a) and the related, NCX4215 [18] are both nitrooxy ester derivatives of aspirin, often referred to as "nitroaspirins". More recently, another series of NO-aspirin hybrid drugs, the furoxan derivatives, which utilise a furoxan NO-donor moiety have been developed [12,19]. These drugs link an NO-donating moiety (furoxan group) by ester linkage to the aspirin molecule (Fig. 1b). Furoxan hybrids of aspirin with NO donor moieties have shown some benefit in avoiding acute gastric injury [12], and to be effective antiplatelet agents [19]. As the compounds have been demonstrated to inhibit COX, they have the potential to retain an aspirin-like anti-inflammatory action. Through their NO release [19], these drugs gain further potential to be anti-inflammatory through the multiple actions of NO [for review see; [20]].

This manuscript explores the impact of two of these novel hybrid compounds and of the furazan analogues, devoid of NO-release capacity (Fig. 1c), on release of the pro-inflammatory cytokine, TNFα from human monocyte-derived macrophages and monocytes. Cytokines are polypeptide or glycoprotein factors that act in an autocrine and/or paracrine fashion to signal in a variety of biological processes. They are generally considered to be either pro-inflammatory (e.g. TNFα and interleukin (IL)-8) or anti-inflammatory [e.g. TGFβ and IL-10] although they can have paradoxical actions. Cytokines act in various cell types and perform diverse functions. TNFα is secreted by monocytes, macrophages and neutrophils following their stimulation by bacterial LPS. The various activities of TNFα include mediation of cell adhesion molecules [21], regulation of cell death in tumour cells [22], enhancement of neutrophil responsiveness [23], control of neutrophil adherence to the endothelium [21] and synthesis of IL-1 production by macrophages [24]. LPS is known to stimulate TNFα production via the activation of the transcription factor, NF-κB [25-27]. It is well known that anti-inflammatory glucocorticoids such as dexamethasone can inhibit release of cytokines such as TNFα from cells stimulated with LPS [28-30]. Possible mechanisms include the ability of glucocorticoids to effectively suppress pro-inflammatory transcription factors such as NF-κB [31] through stimulation of glucocorticoid receptors, which subsequently translocate to the nucleus preventing histone acetylation, a vital step in the NF-κB-induced gene transcription [31,32]. Other mechanisms by which the glucocorticoid receptor reduces TNFα expression include decreasing mRNA stability, inducing expression of the inhibitor IκB, and altering co-factor (AP-1) activity [33]. NO and aspirin have both been previously shown to influence the release of TNFα likely through modulation of NF-κB function [34-42], thus hybrid NO-aspirins may have increased anti-inflammatory potential through the dual action of NO and aspirin moieties on this pathway. Here, we set out to determine if novel NO-releasing furoxan derivatives of aspirin possess anti-inflammatory properties in LPS-activated human monocyte-derived macrophages and monocytes through analysis of TNFα release and the NF-κB pathway. The relative contribution of the aspirin and NO moieties on these effects were also addressed through the use of NO-free furazan counterpart drugs, the NO donor, DEA/NO and aspirin compounds.

## Methods

### Materials

General laboratory supplies were purchased from Sigma (Poole, UK) unless otherwise stated. 2-(*N,N*-diethyamino)-diazenolate-2-oxide (DEA/NO; Axxora, Nottingham, U.K.) was dissolved and stored frozen in 0.01 M NaOH prior to final dilution with phosphate-buffered saline (PBS) immediately prior to use. All NO-aspirin hybrids were synthesized at the Università degli Studi di Torino, as described [12]. They were dissolved in dimethyl sulfoxide (DMSO) then diluted in PBS to give a final DMSO concentration ≤ 0.1%.

### Preparation of Monocytes and Macrophages

Peripheral venous blood was drawn from the antecubital fossa of human volunteers (non-smokers; age 20–45 years). Mononuclear cells were isolated by dextran sedimentation and discontinuous Percoll gradient centrifugation as described [43]. Mononuclear cells were resuspended at a concentration of 4 × 10^6 ^cells/ml in Iscove's DMEM. Monocytes were plated out in 48-well plates at a concentration of 2 × 10^6 ^per well or 6-well plates at 12 × 10^6 ^per well. After 1 h, non-adherent cells were removed by washing wells with Hank's buffered salt solution (HBSS). Macrophages were derived from monocytes by culturing the monocytes in Iscove's DMEM (supplemented with 10% autologous serum) at 37°C for a week, with the medium being changed after 3–4 days [44].

### Stimulation of Cells

For macrophages, on day 7, the medium in each well was changed and fresh medium containing 10 μM test drug or a DMSO vehicle control added to wells with or without the inflammatory stimulant, LPS (10 ng/ml). Drug concentrations were selected based on results from pilot studies and on realistic plasma concentrations, where these data are available; 10 μM was deemed a relevant plasma level for aspirin, and so was utilised in this study. In order to facilitate direct comparisons, the effects of the hybrid compounds were also investigated at 10 μM. Drugs investigated were the furoxan-aspirin hybrids (3-cyanofuroxan-4-yl)methyl 2-acetoxybenzoate (B8) and (3-carbamoylfuroxan-4-yl)methyl 2-acetoxybenzoate (B7), their respective NO-free counterparts (furazans), (4-cyanofurazan-3-yl)methyl 2-(acetoxy)benzoate (B16) and (4-carbamoylfurazan-3-yl)methyl 2-acetoxybenzoate (B15), nitroaspirin NCX4016, NO donor, DEA/NO or dexamethasone (1 μM). Cells were then incubated at 37°C for 4 h before removal of the cell supernatants, which were subsequently frozen at -70°C for future studies. The same procedure was also carried out on monocytes that had not been matured into macrophages with drug treatments and 4 h incubations taking place immediately after the final cell washing step of the isolation procedure.

### Enzyme-Linked Immunosorbent Assay (ELISA)

A human TNFα ELISA kit was purchased from BD Biosciences (cat no; 550610) and performed as per kit instructions on the supernatants removed from macrophage or monocyte cultures. The absorbance of each well was read at 450 nm using a Thermo Labsystems Multiskan Ascent plate reader running Ascent software Version 2.6.

### Lactate Dehydrogenase (LDH) Assay

The cytotoxic impact of the compounds was assessed by measuring release of the enzyme LDH in the supernatant using a kit purchased from Roche (cat no. 1 644 793).

### Western blotting for IκBα

Using 6-well plates, mononuclear cells were prepared as above. Following washing, cells were treated with Iscove's DMEM supplemented with 10% autologous serum containing either B8 (1 μM, 10 μM, 20 μM, 100 μM), gliotoxin (0.1 μg/ml), buffer or a DMSO vehicle control (0.2%) for 30 min at 37°C. LPS (10 ng/ml) or medium was then added to appropriate wells and left to incubate for a further 45 min as described for the immunofluorescence experiment (see below). Lysates were prepared at 4°C using a protease inhibitor cocktail in TBS with 1% Nonidet P40 in order to minimise proteolysis problems. An aliquot of each lysate was used for total protein determination using a BCA protein assay kit (Pierce, Rockford, IL) and an equivalent of 24 μg of protein per well was run on a 12% SDS-PAGE gel and transferred to PVDF. Blots were blocked with 5% skimmed milk in TBS/0.1% Tween-20 before probing with rabbit IκB-α (AbCam, cat no. 32518-100) [45] diluted 1:2500, incubated overnight at 4°C. Subsequently, the blots were washed and incubated with goat anti-rabbit HRP (DakoCytomation, cat no. P0448), also diluted 1:2500, and developed using standard ECL (GE Healthcare).

### Immunofluorescence for NF-κB p65 Subunit

Immunofluorescence for the p65 subunit was used to visualise the translocation of NF-κB. Mononuclear cells were isolated and resuspended as described above. 4 × 10^6 ^(1 ml) cells were placed on a glass coverslip within a 6-well plate and left in an incubator (37°C; 1 h) to adhere. Non-adherent cells were then removed by washing wells with HBSS. Following washing, medium was changed to Iscove's DMEM (supplemented with 10% autologous serum) containing either gliotoxin (0.1 μg/ml), B8 or B16 (20 μM) or no drug (DMSO 0.1% control). Cells were left to incubate (37°C) for 30 min. Drug concentration and incubation times were chosen from pilot studies that demonstrated them to give optimum results in this system. LPS (10 ng/ml) or vehicle (Iscove's DMEM supplemented with 10% autologous serum) was then added on top of the coverslip and left to incubate for a further 45 min. The cell-covered coverslips were then washed 3 times with PBS and 1 ml of 3% paraformaldehyde (in deionised water; dH_2_O) was added and the cells left to fix (RT) for 20 min. Coverslips were then washed a further 3 times with PBS and subsequently incubated for 10 min at RT with 1 ml of 50 mM glycine to quench any aldehyde groups. Following another 3 PBS washes, 1 ml of blocking solution (10% sheep serum in 0.2% fish skin gelatin (Sigma) was added to the coverslips and they were left overnight at 4°C. The following morning, after washing the cells, 100 μl of primary antibody (NF-κB p65 mouse anti-human, BD Biosciences cat no. 610868 [46]; 1:50 dilution in blocking solution) was added to the cells. 1 h later, following three PBS washes, 100 μl of a 1:250 dilution (in blocking solution) of secondary antibody (Alexa Fluor^® ^488 goat anti-mouse IgG, Invitrogen cat no. A-11001) was added and left to incubate for 1 h at RT in the dark. Finally, three PBS washes, followed by three dH_2_O washes were carried out to prevent crystal formation. Coverslips were then mounted onto slides using Moviol (Calbiochem, Merck, Nottingham, UK) and the slides were then stored in the dark at 4°C. Images from the immunofluorescence slides were captured with a camera connected to a Zeiss Axiovert S100 microscope using Improvision Openlab 3.1.5 software. Images were captured at a magnification of ×100.

### Statistical analyses

Statistical analysis was by 1-way analysis of variance (ANOVA) with Dunnett's post-test where applicable and was performed using GraphPad Prism version 4 (GraphPad Software, San Diego, USA). ** is used to represent a P value < 0.01, * denotes a P value < 0.05 and P values greater than 0.05 were deemed not significant. Where expressed, data are in the form mean ± standard error of the mean (S.E.M.).

## Results

### Effect on NO-furoxans on TNFα Release

**Figure 2:**
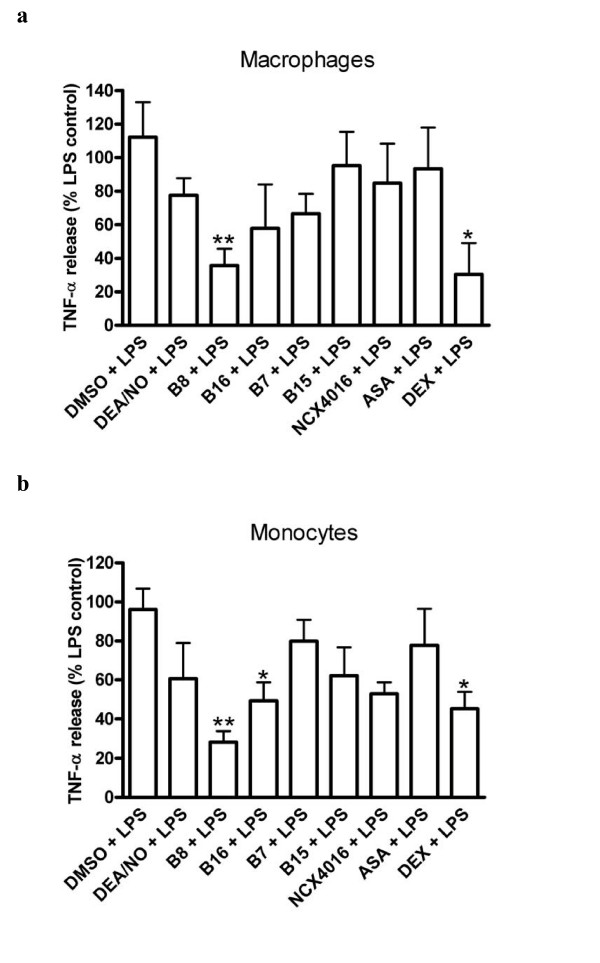
**Effect of potential anti-inflammatory agents on LPS (10 ng/ml)-induced TNFα release in human (a) monocyte-derived macrophages and (b) monocytes after 4 h treatment with 10 μM of either DEA/NO, B8, B16, B7, B15, NCX4016 or aspirin or 1 μM of dexamethasone (Dex)**. For macrophages n = 5–10 and for monocytes n = 4–6 separate donors. ** = p < 0.01 * = p < 0.05 determined by One-Way ANOVA followed by Dunnett's test.

B8 had a significant inhibitory effect on TNFα release in human monocyte-derived macrophages treated with LPS (36 ± 10% of LPS control, P <0.01; n = 5–10 separate donors, Fig. 2). The effect was equivalent in magnitude to that of dexamethasone, but was not shared by DEA/NO, B7, the furazans, aspirin or NCX4016. In monocytes, B8, and to a lesser extent, its NO-free equivalent, B16, significantly inhibited TNFα release [to 28 ± 5% (P < 0.01), and 49 ± 9% (P < 0.05) of control respectively, n = 4–6). Basal TNFα levels were 0.9 ± 0.2 pg/ml for macrophages and 1.6 ± 0.4 pg/ml for monocytes. After LPS-treatment these rose to 5.5 ± 0.5 and 9.6 ± 0.3 ng/ml respectively.

### Effect on NO-furoxans on LDH Release

**Figure 3:**
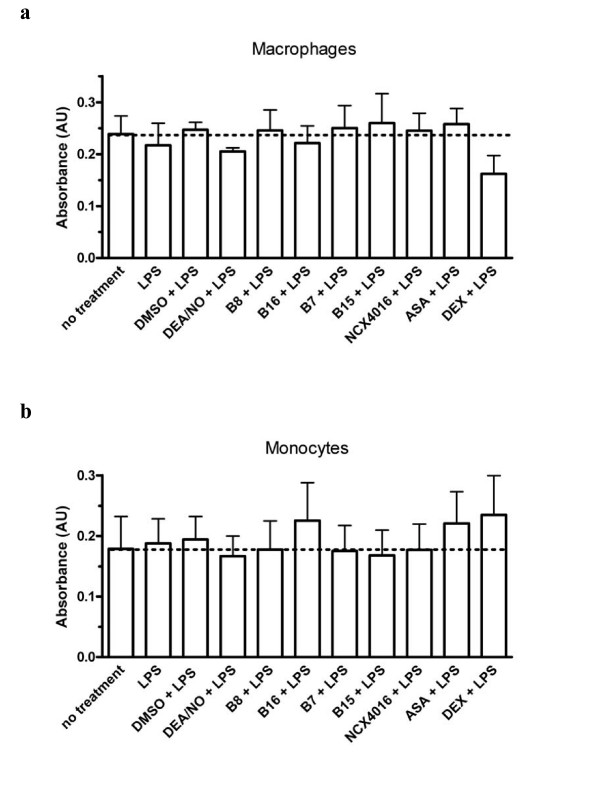
**Bar graphs show LDH measurement in (a) monocyte-derived macrophages and (b) monocyte supernatants after treatment with 10 μM of either DEA/NO, B8, B16, B7, B15, NCX4016 or aspirin or 1 μM of dexamethasone (Dex) and stimulated with LPS (10 ng/ml)**. For macrophages n = 5–15 and for monocytes n = 6 separate donors. One-way ANOVA revealed that there were no significant differences between groups in either cell type.

None of the treatments studied caused significant cell death (Fig. 3) compared with untreated macrophages and monocytes. Levels of LDH released following the treatments were comparable with that from untreated control samples (~0.5 × 10^5 ^cells/treatment).

### Effect on NO-furoxans on NF-κB activation

**Figure 4:**
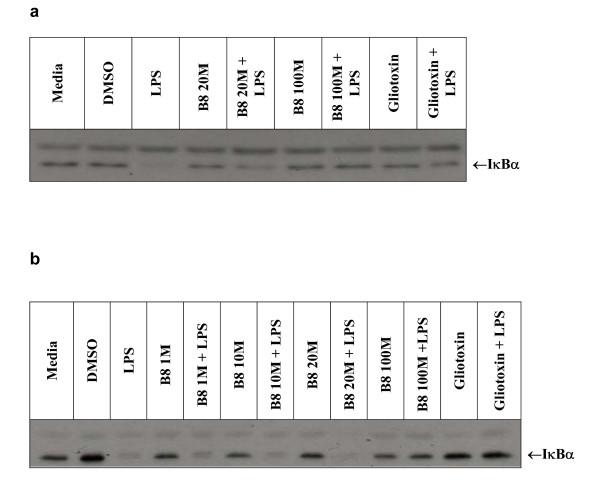
**Western blots showing the effects of varying concentrations of B8 on *IκB*α**. Mononuclear cells were prepared as per methods, plated on 6-well plates, allowed to adhere for 1 h then treated with varying concentrations of B8 (1 μM, 10 μM, 20 μM, 100 μM), gliotoxin (0.1 μg/ml), buffer or a DMSO vehicle control (0.2%) for 30 min at 37°C. After this interval LPS (10 ng/ml) or buffer was added to appropriate wells and left to incubate for a further 45 min. Lysates were prepared, total protein determined and 24 μg of protein per well was run on a 12% SDS-PAGE gel, transferred to PVDF. Blots were blocked before probing with rabbit IκB-α diluted 1:2500, incubated overnight at 4°C. Subsequently, the blots were washed and incubated with goat anti-rabbit HRP, also diluted 1:2500, then developed using standard ECL. Blots are representative of at least 6 similar experiments.

**Figure 5:**
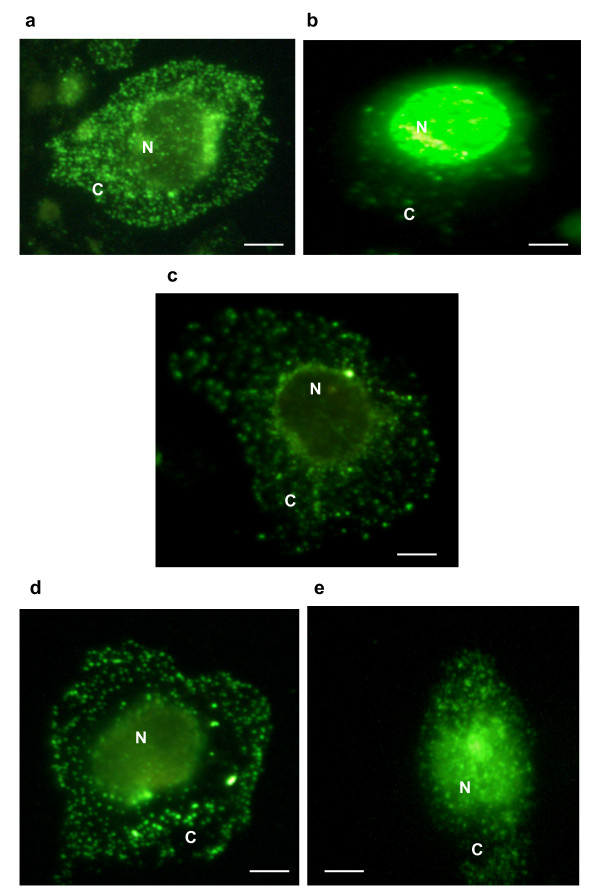
**Representative immunofluorescence images.** Image **a **shows a control treated monocyte. Image **b **shows a monocyte following a 45 min LPS stimulus. Image **c **shows a monocyte treated with gliotoxin (0.1 μg/ml, 30 min) and stimulated with LPS (10 ng/ml, 45 min). Image **d **shows a monocyte treated with B8 (20 μM, 30 min) and stimulated with LPS (10 ng/ml, 45 min). Image **e **shows a monocyte treated with B16 (20 μM, 30 min) and stimulated with LPS (10 ng/ml, 45 min). C = cytoplasm. N = nucleus. Scale bar represents 100 μm. Similar images were obtained on at least 3 separate experiments.

In order to investigate the potential molecular mechanism of action of the inhibitory effect of B8 on macrophage TNF-α release we investigated the effect of the compound on LPS-stimulated NF-κB activation. For this we assessed the effect of B8 on the loss of the cytoplasmic inhibitory subunit of NF-κB, IκBα (Fig. 4a and 4b shows two typical western blots of cytoplasmic IκBα). In both blots LPS causes a dramatic loss of cytoplasmic IκBα, an effect that was completely inhibited by the NF-κB inhibitor gliotoxin. Similarly, B8 at 100 μM also blocked LPS-induced loss of cytoplasmic IκBα (Fig. 4a and 4b) whereas 1 and 10 μM B8 (Fig. 4b) did not affect LPS loss of IκB. The concentration of B8 that appears to be on the threshold of inhibition appears to be around 20 μM (Fig. 4a). In order to confirm that B8 inhibits NF-κB activation more directly, we used immunofluorescence to investigate its effect on LPS stimulation of NF-κB p65 subunit translocation from the cytoplasm to the nucleus. NF-κB p65 immunofluorescence revealed that the subunit location varied according to the drug treatment. Control cells displayed uniform staining throughout the cytoplasm, but following stimulus with LPS, strong staining was observed in the nucleus and much less in the cytoplasm (Fig. 5a, b). Incubation with gliotoxin before the LPS stimulus inhibited the nuclear translocation of p65 as demonstrated by the presence of cytoplasmic staining (Fig. 5c). Pre-incubation with the NO-aspirin B8 gave a dramatic shift in the staining compared to the LPS control. Location of the NF-κB p65 subunit was now revealed to be cytoplasmic (Fig. 5d). Cells treated with the NO-free equivalent of B8, B16, displayed cytoplasmic staining but with a greater amount of nuclear staining than B8 treated cells (Fig. 5e).

## Discussion

### Impact of NO-furoxans on TNFα-Release by Macrophages and Monocytes

The NO-aspirin, B8, had a significant inhibitory effect on TNF-α release from human monocyte-derived macrophages treated with LPS. In monocytes B8, and to a lesser extent, its NO-free equivalent, B16 again caused significant inhibition of TNF-α release. In monocyte-derived macrophages, the lack of effects of B16 and aspirin suggest that the inhibitory effect of B8 on TNFα release is NO-mediated. However, as this effect is not mimicked by the NO-donor, DEA/NO, it is apparently a specific property of B8 that is possibly related to amount, duration or site of NO release. The possibility of diminished release of TNFα by B8-treated cells being simply due to a cytotoxic effect of the compound was not supported by results from the LDH assay. None of the treatments significantly affected cell death when compared with untreated cells.

In monocytes, TNFα release was inhibited by the NO-aspirin, B8, but also by its NO-free furazan counterpart, B16. Aspirin showed no significant difference from the LPS control. Similar to the macrophage results, DEA/NO did not cause significant inhibition. Again, B8 could be acting via an NO-mediated mechanism specific to the amount, duration or site of NO release. However, the interesting observation that NO-free, B16 also causes a significant inhibition suggests a possible further mechanism. As previously demonstrated, the acetyl group of these compounds is lost in plasma [12], leaving salicylic acid, through which, inhibition of cytokine release has been reported [47-49]. It may therefore be possible that under these experimental conditions, a salicylic acid-mediated mechanism is responsible for the inhibition of TNFα release observed with B8- and B16-treated cells. It is probable that the result obtained with B8 is achieved through combination of the effects of NO (as illustrated by DEA/NO) and that of the NO-free B16. We have previously shown that the furoxan-aspirin B7 releases significantly less NO than B8 [19] and thus suggest that the NO release from B7 is insufficient to result in an anti-inflammatory effect similar to that of B8. Our result obtained with B16 indicates that altering the chemical structure of aspirin clearly has an impact on the ability to achieve anti-inflammatory properties in this assay. Furthermore, the alteration to the aspirin structure in B15 and B7 likely contributed to their poor performance in this assay.

In this study, aspirin did not significantly reduce TNFα release from LPS-stimulated monocytes or macrophages. This is consistent with a similar study in which aspirin failed to have an effect even at a 30-fold higher concentrations than was used in the present study [50]. A further study did report an inhibitory effect of aspirin on TNFα release from LPS-stimulated monocytes but this was at concentrations of 5–10 mM [49], which may not be representative of pharmacologically relevant plasma concentrations [51]. We show here that NCX4016 did not significantly reduce the release of TNFα. In a study carried out by others, NCX4016 did not inhibit TNFα release at the same concentration used here (10 μM), but was shown to inhibit the release of TNFα and IL-6 from LPS-stimulated macrophages at higher (100 and 300 μM) concentrations and following a 6 h incubation [50]. Despite not being affected by the soluble guanylate cyclase inhibitor ODQ, the authors suggest that the inhibitory effect of NCX4016 is NO-mediated due to the failure of aspirin to inhibit cytokine release. A further study also showed that NCX4016 (again at concentrations 10-fold higher than used in this study), inhibited the release of IL-1β and IL-18 from LPS-stimulated monocytes, via NO-mediated inhibition of the enzyme required for intracellular processing and maturation of IL-1 and IL-18 (caspase-1) activity [52]. It is likely that the differing outcomes observed between this and other studies are purely due to drug incubation time or concentrations. The concentration studied here is based on the realistic relevant plasma concentrations of aspirin [51] and so is more demonstrative of the true therapeutic potential of the drug. The results here show that at a concentration at which the furoxan compound, B8, causes a significant 72% reduction in TNFα release from monocytes and a 64% reduction from macrophages, its organic nitrate counterpart does not.

Possible explanations for the differential effects of the same treatments observed between monocytes and macrophages effect may be the differential expression and activity of receptors and signalling pathways between the two cell types. It has previously been reported that the anti-inflammatory effect of IL-4 on the release of TNFα and other cytokines, varies between LPS-stimulated monocytes and macrophages [53]. This effect is due to loss of a receptor for IL-4 during monocyte differentiation [53]. Other differences reported between monocytes and macrophages include those showing that LPS activates cytosolic PLA_2 _in monocytes but not in macrophages. A similar activation in monocytes, but not macrophages, is seen after LPS-stimulation in the following signalling pathways: the MAP kinase, ERK, phosphatidylinositol-3 kinase and p70S6 kinase [54,55]. Further variations include increased expression of Ca^2+^-dependent protein kinase C isoforms in monocytes when compared to macrophages [56] and also that maturation into macrophages results in slower production of the cytokine, IL-1β [57]. It is, therefore, possible that changes such as these to receptor expression, signalling pathways and to the biosynthesis of cytokines, which normally occur during the maturation of monocytes with macrophages, may impact on the ability of the studied compounds to have a significant effect.

### Impact of NO-furoxans on NF-κB Activation in Monocytes

The western blot and immunofluorescence experiments further suggested a possible mechanism for the B8-mediated inhibition of TNFα release. The transcription factor, NF-κB, exists as a dimer (can be either a homodimer or heterodimer). The most common form is a heterodimer composed of the p65/p50 subunits [58]. Normally, NF-κB is kept in an inactive state in the cytoplasm, bound to an inhibitory subunit named IκBα [59]. The phosphorylation of IκBα by a kinase known as IKK and the subsequent degradation of IκBα leads to the activation of the NF-κB [60]. The NF-κB dimer containing the p65 subunit; the dominant factor in the induction of the TNFα gene, then translocates from the cytoplasm to the nucleus where it activates transcription of target genes such as TNFα [25,27,61,62]. To investigate the effect of the NO-aspirin on stimulus (LPS)-induced NF-κB activation, we used two independent assays; namely loss of cytoplasmic IκBα assessed by western blotting and translocation of the p65 subunit of NF-κB assessed by immunofluorescence. For consistency, LPS was used as our NF-κB-activating stimulus and epipolythiodioxoperazine (gliotoxin), a known immunosuppressive agent that inhibits NF-κB activation by preventing IκBα degradation [43,63], was used as the positive control. LPS caused a dramatic loss of cytoplasmic IκBα and translocation of cytoplasmic p65 to the nucleus, effects that were inhibited by NO-aspirin B8 and the positive control gliotoxin. Thus, the inhibition of NF-κB activation provides a plausible explanation for the B8-induced reduction in TNFα release as observed in the ELISA studies. It has been shown that NO inhibits LPS-induced IκB-phosphorylation and inhibits the activation of NF-κB [35], further supporting our paradigm of NO-mediated inhibition of TNFα release by B8. B16-treated and LPS-stimulated monocytes also displayed some evidence of cytoplasmic staining, but with more nuclear staining than its NO counterpart. This result is consistent with the ELISA data, where significant inhibition of TNFα release, albeit lesser than B8-treated cells, was observed following B16 treatment.

### Therapeutic Implications

**Figure 6:**
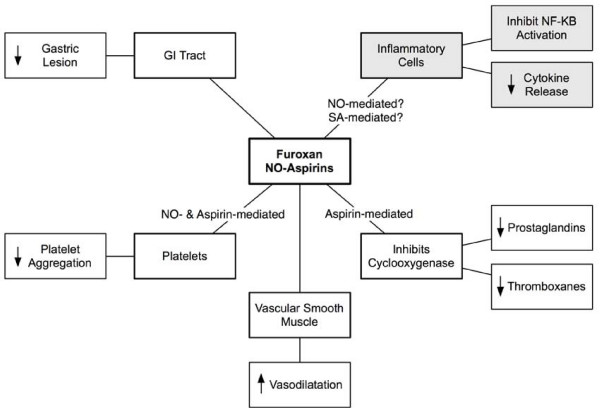
**A schematic representation of the effects and potential uses of furoxan-aspirin hybrid drugs**.

The data here show that the furoxan-aspirin compound B8 has anti-inflammatory effects in LPS-stimulated monocytes and macrophages through its reduction in NF-κB-mediated TNFα release. This action of B8 may be useful in inflammatory diseases (arthritis, Crohn's disease and asthma [64-67] where anti-TNFα therapy is, or has potential, to be of therapeutic benefit. Rheumatoid arthritis is a chronic inflammatory autoimmune disorder characterised by inflammation of the lining (synovium) of joints. Joint deterioration, together with the pain associated with synovial inflammation can lead to substantial loss of mobility. Pro-inflammatory cytokines are abundant in the joints of sufferers [65]. Anti-TNFα drugs have been recently licensed for use in arthritis in a bid to limit the contribution of this cytokine to the chronic joint inflammation [67]. Treatment of arthritis with drugs of the NSAID class is severely limited due to the gastric side-effects associated with the high doses and chronic nature of the treatment required. However it is hoped that NO-aspirins might offer a preferable alternative to therapy with conventional NSAIDs. The multifaceted actions of B8 on COX inhibition [19] the anti-TNFα effects demonstrated here, its observed resistance to gastrotoxic effects [12] and its potential for targeted intracellular release of NO [19,68] could indicate B8 to be a promising anti-arthritic drug. A summary of the effects of the furoxan-aspirin hybrid drugs is provided in Fig. 6. A further, more speculative application for drugs such as B8 is in atherosclerosis therapy. It is now widely accepted that inflammation is a key element in atherogenesis and atherosclerotic plaque rupture leading to acute cardiovascular events such as myocardial infarction or stroke [69]. The release of TNFα by monocytes and macrophages causes various effects involved in destabilising the atherosclerotic plaque [70-74]. Such evidence may suggest that drugs with an anti-TNFα action, such as B8, may be of benefit in atherosclerosis.

## Conclusion

Taken together, these studies provide evidence that treatment with NO-aspirin B8, significantly reduced TNFα release from both LPS-stimulated monocytes and monocyte-derived macrophages. A possible mechanism for this anti-inflammatory action is through the inhibition of its transcription factor, NF-κB by NO. Such an action instils a potential for drugs of this NO-aspirin hybrid class to be utilised as anti-inflammatory agents for the treatment of a wide range of inflammatory conditions such as arthritis.

## Abbreviations

ANOVA: Analysis of variance; COX: Cyclooxygenase; DEA/NO: 2-(*N,N*-diethyamino)-diazenolate-2-oxide; dH_2_O: deionised water; DMSO: Dimethyl sulfoxide; ELISA: Enzyme-linked immunosorbent assay; HBSS: Hanks buffered salt solution; IL: Interleukin; LDH: Lactate dehydrogenase; LPS: Lipopolysaccharide; NO: Nitric oxide; NSAID: Nonsteroidal anti-inflammatory drugs; NF-κB: Nuclear factor-kappa B; PBS: Phosphate-buffered saline; RT: Room temperature, TNFα: Tumour necrosis factor-alpha.

## Competing interests

The authors declare that they have no competing interests.

## Authors' contributions

The manuscript was written and the experiments were designed by CMT and AGR. CMT performed the TNF-α release, LDH assay and immunofluorescence experiments, PM performed the western blot experiments and TAS performed and assisted in all the experimental procedures. Hybrid drugs were synthesised and supplied by LL, CC, RF and AG. AGR and ILM supervised the experiments and oversaw manuscript construction together with SF, revising it critically for important intellectual content. All authors have given final approval of the version to be published.
